# Double Thyroglossal Duct Cyst: A Case Report

**DOI:** 10.7759/cureus.40660

**Published:** 2023-06-19

**Authors:** Jeremy Walsh, Sean Clausen, Jason Degiovanni, Michele M Carr

**Affiliations:** 1 Otolaryngology, University at Buffalo Jacobs School of Medicine and Biomedical Sciences, Buffalo, USA

**Keywords:** sistrunk procedure, rare head and neck, thyroid embryology, pediatric neck masses, thyroglossal duct cyst

## Abstract

Thyroglossal duct cysts are one of the most common cervical congenital anomalies. They occur along the thyroid migration tract, which extends from the base of the tongue through the midline of the neck to the level of the cricoid cartilage. Thyroglossal duct cysts present as a midline neck mass and are closely associated with the hyoid bone. Here, we describe a case where two cystic structures were found just inferior to the thyroid gland and inferior to the hyoid bone, suggesting a double thyroglossal duct cyst. It is important to diagnose and surgically manage thyroglossal duct cysts as they are associated with complications, such as infection and malignancy.

## Introduction

Thyroglossal duct cysts (TGDC) are one of the most common congenital anomalies seen in the pediatric population. As the thyroid gland begins to develop at the foramen cecum, it descends into the neck through the thyroglossal duct. Around the 10th week of gestation, the thyroglossal duct involutes [1]. Failure of involution of the thyroglossal duct at any location can give rise to TGDC formation [1]. This study describes a rare occurrence of a double thyroglossal duct cyst, which has only been documented in a handful of cases in the existing literature.

## Case presentation

**Figure 1:**
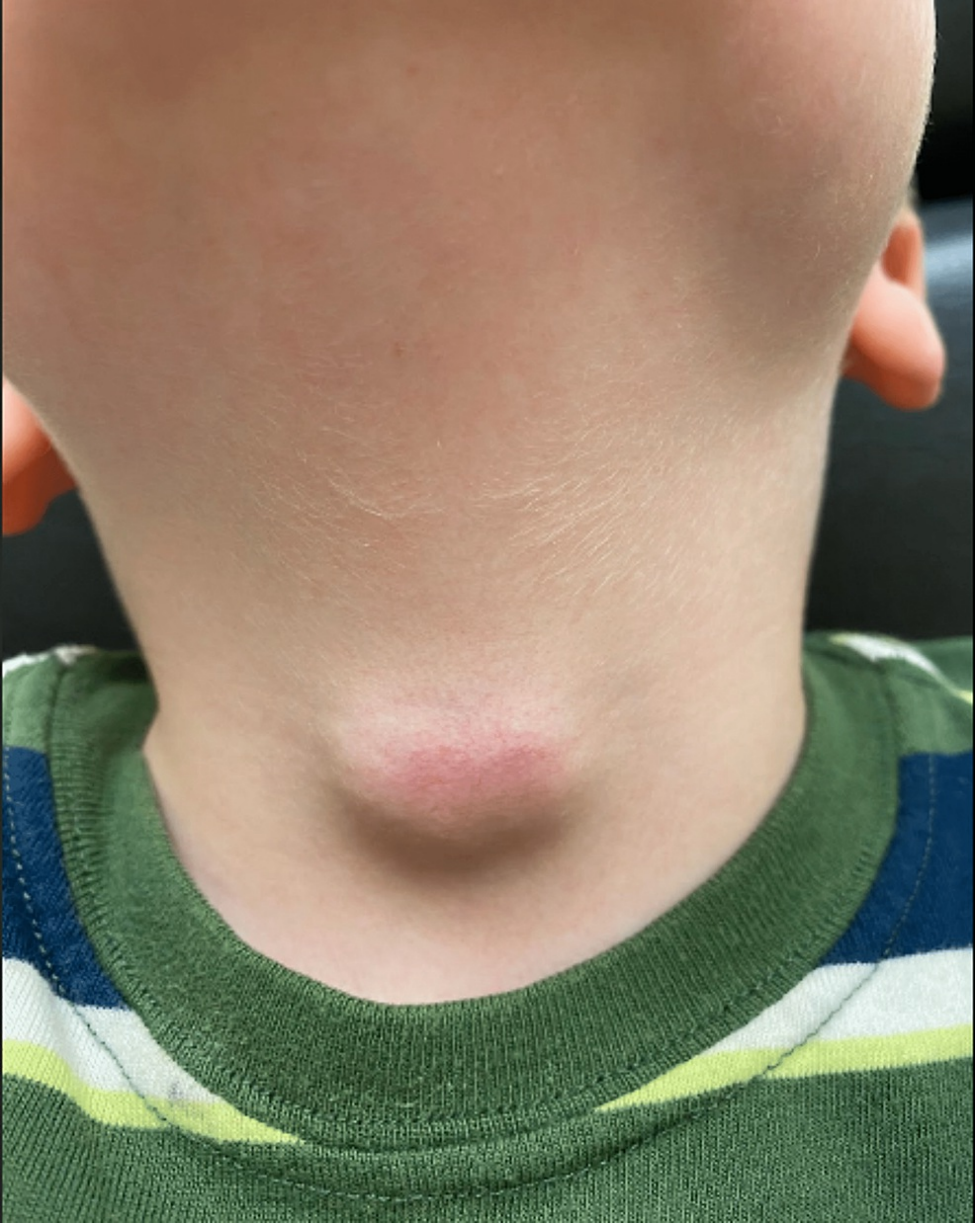
Erythematous anterior midline neck mass.

A five-year-old boy was seen in our clinic for an evaluation of a midline anterior neck mass. One week prior to presentation, the mass was first noticed, and at the time of assessment, the only reported symptom was neck tenderness. Physical examination was otherwise unremarkable. Two oval masses, measuring approximately 3×2 cm were palpated in the midline of the neck with erythema overlying the inferior mass (Figure 1).

**Figure 2:**
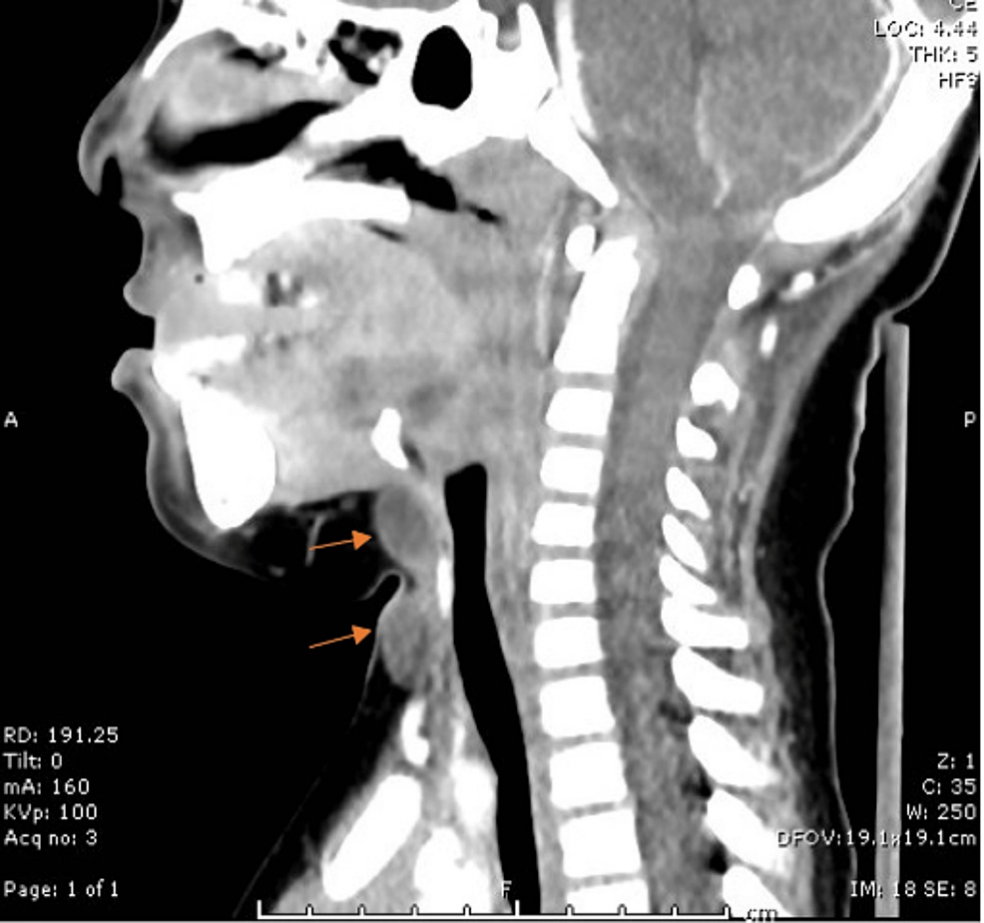
CT scan reveals two, well-defined smooth-bordered oval homogeneously appearing lesions located inferior to the hyoid bone and thyroid gland.

No cervical lymphadenopathy was present. An ultrasound revealed two hypoechoic lesions in the anterior neck, one measuring 1.6×1.0×1.9 cm and the other one measuring 1.2×0.8×1.2 cm. The radiology report suggested a bilobed thyroglossal duct cyst. He was given antibiotics for a suspected cyst infection. One month after he originally presented to the clinic, he returned with resolved erythema but complained of residual discomfort in the area. Computed tomography with contrast was performed and revealed two midline neck masses (Figure 2). There was a well-defined 1.7×1.7×1.0 cm, non-rim enhancing, low attenuation mass inferior to the thyroid gland. Another well-defined 1.2×1.1×1.6 cm, non-rim enhancing, low attenuation mass inferior to the hyoid bone/superior to the isthmus.

The patient underwent surgery using the Sistrunk procedure, resulting in the complete removal of the cysts through one inferior incision. During the dissection, no obvious tract was identified. A cuff of midline tissue was taken between the cystic structures. Surgical pathology concluded that the tissue was heavily inflamed fibroconnective tissue with fragments of hyoid bone. There was no cystic lining or thyroid tissue identified. These findings were compatible with disrupted and inflamed thyroglossal duct cysts.

## Discussion

Thyroglossal duct cysts (TGDC) are one of the most common congenital anomalies, presenting in about 7% of the population [1]. Although most cases of TGDC are recognized by the age of five years, as seen in our patient, roughly 60% of cases will present before the age of 20 years [2]. TGDC typically presents as a mobile and painless mass located in the midline of the neck. Vertical movement of the mass with swallowing and tongue protrusion, along with the mass’s close proximity to the hyoid bone, is highly indicative of TGDC. They are usually asymptomatic and can be found incidentally on physical examination, however, may result in fever, erythema, tenderness, dysphonia, and dysphagia [3]. Further diagnostic imaging should be obtained to rule out other midline neck masses including dermoid cyst, ectopic thyroid tissue and malignancies, branchial cleft cyst, lymphadenopathy, lymphangioma, lipoma, and teratoma [4,5]. Ultrasonography (US), magnetic resonance imaging (MRI), computed tomography (CT) and thyroid scintigraphy can all be used to confirm the diagnosis. In this case, an US was ordered before referral to our office resulting in suspicion of TGDC, even though it was bilobed. CT did not confirm the diagnosis but narrowed our differential diagnosis. Furthermore, the CT revealed normal thyroid tissue. There is controversy about preoperative radioisotope thyroid scanning in patients with presumed TGDC. While some argue for routine scanning to identify ectopic thyroid tissue, which can result in permanent hypothyroidism if excised, the incidence of such tissue found during surgery ranges from 0.5% to 5.7% [6]. With careful consideration, we decided against obtaining blood work or specific thyroid imaging prior to the procedure.

Surgical management is the only definitive treatment option for TGDC. The Sistrunk procedure is considered the gold standard treatment as it has been shown to be associated with a lower recurrence rate compared to a simple excision (6.9% vs. 29% recurrence, respectively), presumably because of complete removal of the thyroglossal duct remnants [7]. The Sistrunk procedure generally starts with an anterior neck incision. The superior and inferior subplatysmal flaps are created and the strap muscles are separated at the linea alba. The cyst is located and dissected starting from the inferior surface with caution to avoid cyst rupture. The dissection is then continued superiorly along the presumed track, as one is not usually clearly identified. The strap muscles are then dissected from the hyoid bone and the central portion of the hyoid bone is separated medially to the lesser cornua bilaterally. Dissection continues to the musculature towards the foramen cecum. The presumed tract in the oropharynx is ligated with a silk tie. The cyst, central hyoid bone, and presumed tract are removed en bloc, as seen in our case.

Histology of the TGDC consists of either squamous or respiratory pseudostratified epithelium. They are often filled with mucus, which is contrasted to dermoid cysts that contain keratinaceous debris [8]. The presence of thyroid gland tissue on histology also confirms TGDC, with incidence of ectopic thyroid tissue varying from 31% upwards to 62% in some cases [9]. Correctly identifying the histology is important as it determines the diagnosis and the amount of dissection required. Pathology confirmed our suspicion of a double thyroglossal duct cyst that did not contain cystic lining or thyroid tissue.

According to our literature search, our findings include this case report, among seven others (Table 1), that describe a double thyroglossal duct cyst [2,4-5,10-13]. As anomalies can happen anywhere along the descending tract, there have been common patterns present within the other seven cases. For example, four out of the seven cases reported development of lingual cysts [2,4,5,12]. Also, three of the seven cases had a communicating tract between the two cysts, unlike in our patient who had two disrupted cystic structures [4,10,11]. To our knowledge, this is the first case that has been reported describing the location of double TGDC to be inferior to the thyroid gland and inferior to the hyoid bone.

**Table 1:** Case reports reporting the location of double thyroglossal duct cysts.

Case Reports	Location of cysts
Sarmento et al. (2013) [2]	Superior to geniohyoid and sublingual gland
Yorgancılar et al. (2011) [4]	Cervical mass and tongue base with communicating tract
Yildiz et al. (2014) [5]	Superior to thyroid isthmus and suprahyoid/root of the tongue
Guo et al. (2008) [10]	Suprahyoid and tongue base
Pueyo et al. (2023) [11]	Infrahyoid and left intrathyroid with a communicating tract
Lee et al. (2017) [12]	Infrahyoid and tongue base
Khadivi and Ardekani (2010) [13]	Two separate infrahyoid cysts with communicating tract

## Conclusions

Double thyroglossal duct cysts are extremely rare. Given the rarity of this anomaly, misdiagnosis as a single TGDC with a concomitant different cystic lesion is possible. Imaging and histopathological testing must be performed to confirm the diagnosis of TGDC. Once diagnosed, the Sistrunk procedure is the appropriate treatment to minimize recurrence and complications.
